# Neutrophil-to-lymphocyte Ratio is Associated with LV Diastolic Dysfunction in the Overt Hyperthyroid Patients

**DOI:** 10.3389/fendo.2022.906947

**Published:** 2022-07-14

**Authors:** Huan Zhang, Jiaoyue Zhang, Huan Li, Yaqiong Bi, Linfang Wang, Yuming Li

**Affiliations:** ^1^ Department of Endocrinology, Union Hospital, Tongji Medical College, Huazhong University of Science and Technology, Wuhan, China; ^2^ Hubei Provincial Clinical Research Center for Diabetes and Metabolic Disorders, Wuhan, China; ^3^ Department of Ultrasound, Union Hospital, Tongji Medical College, Huazhong University of Science and Technology, Wuhan, China; ^4^ Department of Gastrointestinal Surgery, Union Hospital, Tongji Medical College, Huazhong University of Science and Technology, Wuhan, China

**Keywords:** hyperthyroidism, left ventricular diastolic dysfunction, neutrophil-to-lymphocyte ratio, prevalence, echocardiography

## Abstract

**Background:**

Recent studies have shown that the neutrophil-to-lymphocyte ratio (NLR) has gradually been identified as a more reliable marker of inflammation, with predictive value for the development of many diseases. However, its association with left ventricular (LV) diastolic dysfunction in overt hyperthyroid patients is unclear. Here, we aimed to explore the relationship between NLR and LV diastolic dysfunction in overt hyperthyroid patients.

**Methods:**

For this study, we retrospected the consecutive medical files of 350 overt hyperthyroid patients. Their medical data and laboratory findings were recorded. According to the presence or absence of LV diastolic dysfunction, the patients with overt hyperthyroidism were divided into two groups. One group with LV diastolic dysfunction included 104 patients and another group with non-LV diastolic dysfunction included 246 patients. The NLR values between the two groups were compared, and the relationship between NLR levels and the prevalence of LV diastolic dysfunction was also explored.

**Results:**

The NLR value in LV diastolic dysfunction group in the overt hyperthyroid subjects was significantly higher than that in non-LV diastolic dysfunction group [1.100 (0.907-1.580) vs 1.000 (0.761-1.405), P=0.016]. The prevalence of LV diastolic dysfunction in Low- (NLR<0.879), Medium- (0.879< NLR<1.287), and High- (NLR >1.287) NLR level groups were 20.9%, 32.5% and 35.7% respectively. Moreover, increased NLR is associated with increased prevalence of LV diastolic dysfunction, and after adjustment for potential associated factors, NLR remained significantly associated with LV diastolic dysfunction. (OR = 11.753, 95%CI = 1.938-71.267, P = 0.007).

**Conclusions:**

Our findings demonstrated that the NLR was associated with LV diastolic dysfunction in the overt hyperthyroid patients, and the prevalence of LV diastolic dysfunction may be positively correlated with NLR levels.

## Introduction

Hyperthyroidism, one of the most common endocrine disorders, is reported that the prevalence ranges from 0.2%-1.3% in iodine-sufficient regions of the world (1). Hyperthyroidism can result in a lot of complications, of which cardiovascular disease is the leading cause of life-threatening and death in patients with hyperthyroidism. These may be owing to the receptors on the myocardium and vascular tissue being very sensitive to the changes of thyroid hormone (TH). Small changes in TH concentrations can cause changes in cardiovascular disease physiology (2). In particular, old, overweight or obese patients with overt hyperthyroidism are easily prone to cardiovascular co-morbidities (3–5). In patients with overt hyperthyroidism, changes in left ventricular (LV) load and increased cardiac contractility lead to lead to hyperdynamic circulation that is high at rest but poorly responsive to exertion (6). A previous study has reported that patients with overt hyperthyroidism had an enhanced LV diastolic functions (7). Studies showed congestive heart failure (HF) which is the first clinical manifestation occurs in approximately 6% of patients with overt hyperthyroidism, and half of these congestive HF patients had left ventricular systolic dysfunction (7, 8). HF patients with preserved LV systolic function had better long-term survival than patients with LV systolic dysfunction (9). The prevalence of diastolic HF in the community, without enough attention, is known to be at least as high as that reported in previous studies of hospitalized patients. Approximately 50% of all HF patients had diastolic HF, especially in old women (10). However, the patients with LV diastolic dysfunction in the overt hyperthyroid patients have not received sufficient attention, and there are few data on it. Due to initially asymptomatic in early, late clinical manifestation and missing reliable biomarkers, early detection is challenging and overall survival remains poor. Therefore, it is of great significance to find reliable indicators for early detection and diagnosis of left ventricular diastolic dysfunction in clinical hyperthyroid patients.

Multiple mechanisms might be involved in the pathogenesis of cardiovascular disease in the overt or clinical hyperthyroid patients, and growing studies have revealed that chronic low-grade inflammation induced by metabolic disorders might have a critical role (11). And the neutrophil-to-lymphocyte ratio (NLR) functions as an accurate and reliable index of systemic inflammation (12, 13) receives much attention owing to easy availability and its low cost. It has gradually been identified as a novel and reliable inflammatory markers of cardiovascular prognosis, and a predictor of coronary artery disease severity. It is also of great use for identifying high-risk patients (ie. coronary artery disease, acute coronary syndromes). However, it is unclear whether NLR has a similar predictive effect on the severity of LV diastolic dysfunction in overt hyperthyroid patients, and our study aimed to explore the answer to this question.

## Subjects

This study complied with the Declaration of Helsinki and was approved by the Ethics Committee of Tongji Medical College, Huazhong University of Science and Technology. We reviewed consecutive medical records of inpatients in the Department of Endocrinology, Wuhan Union Hospital, China from May 2018 to May 2019. Our study ultimately included 350 patients who received 131I treatment for overt hyperthyroidism. Subjects who were newly discovered and diagnosed with overt hyperthyroidism and/or who had been off antithyroid drugs for 4 or more weeks were finally enrolled in the analysis. Our research adopted the definitions in the American Thyroid Association(ATA) Guidelines (14), overt hyperthyroidism is defined as simultaneously suppressed thyroid-stimulating hormone (TSH) level, free triiodothyronine (FT3) level, and/or elevated free thyroxine (FT4). The exclusion criteria for this study were set as 1) age <18 years; 2) congenital cardiovascular disorder, valvular heart disease, pericardial disease, cardiomyopathy, infective endocarditis, severe ventricular arrhythmias, heart failure, acute myocardial infarction, pulmonary disease, stroke and anemia with heart disease or cardiac surgery history; 3) with multiple atrial fibrillation (AF); 4) with serious renal failure; 5) lacking blood routine data; 6) acute poisoning, acute hemorrhage, active infection, tissue damage, blood diseases, acute bleeding, active infection, tissue damage, a blood disorder, have a blood transfusion in the past 2 weeks, a medication that affects white blood cell count, immunosuppressive or steroid medication in the past 2 weeks; 7) with carcinoma or malignancy.

## Materials and Methods

### Demographics and Laboratory Results

We reviewed the patients’ medical records and collected their data on age, sex, duration of overt hyperthyroidism, positive family history of thyroid disease, weight, height, body mass index (BMI), systolic blood pressure (SBP), diastolic blood pressure (DBP), neutropenia or agranulocytosis and impaired liver function. BMI was calculated by dividing weight (kg) by the square of height (m). BMI was divided into thin (<18.5 kg/m^2^), normal (18.5–24.0 kg/m^2^), overweight (>24.0 kg/m^2^) (15) in line with the recommended criteria for the Chinese population. Laboratory tests were completed using standard biochemical analytical methods, which included levels of serum uric acid (SUA), serum creatinine (Scr), TSH, FT3, FT4, anti-thyroid peroxidase antibody (TPOAb), anti-thyroglobulin antibody (TgAb), and thyrotropin receptor antibody (TRAb) and blood routine including white blood cell (WBC) count, platelet (PLT) count, neutrophil count, Monocyte count, and lymphocyte count. NLR was calculated as neutrophil count divided by lymphocyte count. Platelet-to-lymphocyte ratio (PLR) was calculated as PLT count divided by lymphocyte count. Impaired liver function is defined as ALT≥40IU/L or AST≥40IU/L.

### Echocardiography

We reviewed the consecutive medical files of the patient’s echocardiographic data. All echocardiographic examinations were performed during hospitalization on enrolled patients adopting echocardiographic systems (GE Vivid 7; Vingmed; Philips IE33 and Philips EPIQ 7C) and 3-8 MHz transducers. Signs of diastolic dysfunction based on the E/A ratio value of the mitral and/or the e/a ratio value of the septal basal regions were performed by two experienced ultrasound doctors. The cutoff value below 1 indicates impaired myocardial relaxation (5, 16). The left ventricular end-diastolic diameter (LVEDD), left atrium diameter (LAD) interventricular septum thickness (IVST), and left ventricular ejection fraction (LVEF) were also recorded. Furthermore, LAD, IVST, and LVEDD were measured from the parasternal LV long-axis view at the level of the mitral valve leaflet tips. LVEF was acquired by the biplane Simpson’s method. Peak velocities in the early (E-wave) (MVE) and late (A-wave) (MVA) phases of the mitral inflow pattern were measured from apical four-chamber views.

### Statistical Analysis

All statistical analyses and graphs were performed with IBM SPSS 24.0 and GraphPad Prism 7.0. Assessing whether a continuous variable is normally or non-normally distributed by the Kolmogorov-Smirnov test. Data that are normally distributed are expressed as mean ± standard deviation (SD), while data that are skewed are expressed as median (25th-75th percentile). Data are categorical and expressed as numbers or percentages as shown. For analysis of the independent variables, the differences between groups were compared using Student’s t-test, Mann-Whitney U test, Kruskal-Wallis test, or chi-square test, as appropriate. Spearman’s correlation analysis was undertaken to explore the associations between thyroid hormone and other clinical parameters. The relationship between NLR and LV diastolic dysfunction was conducted by univariate and multivariate logistic regression analysis. Statistical significance is set at P-values < 0.05.

## Results

### Basic Characteristics and Laboratory Findings of the Overt Hyperthyroid Patient with LV Diastolic Dysfunction

**Figure 1:**
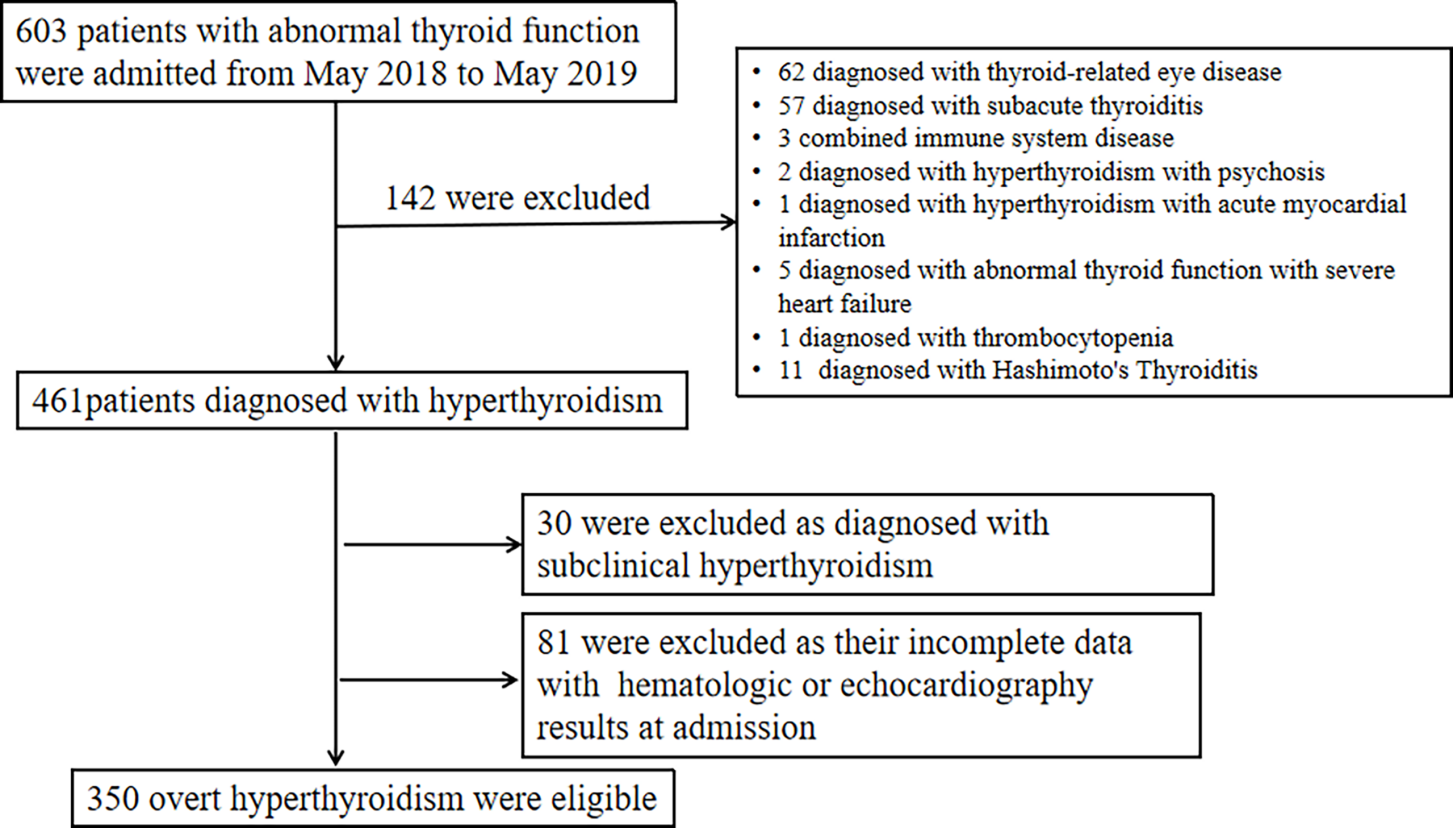
Flow diagram showing the patient selection process.

By May 2019,603 patients with abnormal thyroid function were admitted. After excluding 142 cases diagnosed with other thyroid diseases,30 cases diagnosed with subclinical hyperthyroidism, and 81 cases without hematologic or echocardiography results, a total of 350 individuals with overt hyperthyroidism were included in the analysis (**Figure 1**). A total of 350 overt hyperthyroid subjects were included in this cross-sectional study. The patients were divided into two groups according to the diastolic function, there were 246 overt hyperthyroid subjects without diastolic dysfunction (81 males, 165 females) and 104 overt hyperthyroid subjects with diastolic dysfunction (35males, 69 females). A comparison of all demographic characteristics and laboratory findings of the subjects with LV diastolic dysfunction and non-LV diastolic dysfunction is presented (**Table 1**). Compared with subjects with non-LV diastolic dysfunction, the overt hyperthyroid subjects with LV diastolic dysfunction were older (P < 0.001), had a greater BMI (P < 0.001) and had higher Scr levels (P = 0.029). In addition, LAD, MVA, and IVST were significantly increased in the overt hyperthyroid patients with diastolic dysfunction (all P<0.001). Compared with subjects without diastolic dysfunction, subjects with diastolic dysfunction had significantly lower MVE and E/A ratios (both P<0.001). Lower serum FT3 and FT4 levels were observed in patients with LV diastolic dysfunction, but this difference was of no statistical significance.

**Table 1:** Basic characteristics and laboratory findings of the overt hyperthyroid subjects with LV diastolic dysfunction.

	With non-LV diastolic dysfunction n = 246	With LV diastolic Dysfunction n = 104	P-value
Age (years)	31 (26-43)	47 (40-54)	<0.001
Female, n (%)	165 (67.1%)	69 (67.0%)	0.988
Duration (years)	1.0 (0.08-4.00)	3.0 (0.17-10.0)	0.157
Positive family history thyroid disease (n,%)	47 (19.7%)	19.0 (18.3%)	0.855
Neutropenia/Agranulocytosis (n,%)	92 (37.7%)	38 (36.5%)	0.932
Impaired liver function (n,%)	74 (33.3%)	31 (31.0%)	0.794
BMI (kg/m^2^)	19.8 (18.72-21.68)	22.2 (19.57-24.34)	<0.001
BMI (n)			0.001
thin	51	13	
Normal	170	64	
Overweight	25	27	
SBP (mmHg)	126 (114-135)	129 (120-139)	0.152
DBP (mmHg)	76 (68-83)	74 (71-81)	0.522
Scr (mg/dl)	0.47 (0.38-0.57)	0.50 (0.42-0.65)	0.029
SUA (umol/L)	306 (255-361)	310 (260-358)	0.984
FT3 (pmol/L)	27.8 (16.6-37.6)	22.0 (15.5-35.2)	0.203
FT4 (pmol/L)	66.1 (41.7-100.0)	59.5 (43.3-98.15)	0.410
TSH (mIU/ml)			0.160
<0.005	171 (69.5%)	80 (76.9%)	
≥0.005	75 (30.5%)	24 (23.1%)	
TPOAb (IU/ml)	175.9 (43.5-386.5)	219.0 (50.3-466.9)	0.333
TgAb (IU/ml)	74.6 (13.9-523.7)	177.1 (13.7-1155.0)	0.257
TRAb (IU/L)	10.9 (6.27-25.5)	11.0 (4.1-22.1)	0.738
LAD (cm)	3.1 (2.9-3.4)	3.4 (3.2-3.8)	<0.001
LVEF (%)	65.0 (63.0-68.0)	66.0 (63.0-70.0)	0.276
IVST (cm)	0.9 (0.8-0.9)	0.9 (0.9-1.0)	<0.001
LVEDD (cm)	4.5 (4.3-4.7)	4.6 (4.4-4.9)	0.529
MVE (m/s)	1.0 (0.9-1.2)	0.9 (0.7-1.1)	<0.001
MVA (m/s)	0.7 (0.6-0.9)	0.9 (0.8-1.1)	<0.001
E/A ratio	1.3 (1.2-1.6)	0.9 (0.785-1.2)	<0.001

Data are mean (SD), number (%), or median (25th–75th percentiles) according to the data type and distribution.

In the comparison of leukocyte-derived inflammatory parameters, subjects with LV diastolic dysfunction had significantly lower PLT, monocyte, and lymphocyte counts (both P <0.05). Compared with those without LV diastolic dysfunction, subjects with LV diastolic dysfunction had higher NLR (P = 0.016) (**Table 2**).

**Table 2:** White blood cells-derived inflammatory parameters of the hyperthyroid patients with LV diastolic dysfunction.

	With non-LV diastolic dysfunction N = 246	With LV diastolic dysfunction N = 104	P-value
WBC count (10^9^/L)	4.837 ± 1.732	4.633 ± 1.290	0.228
PLT count (10^9^/L)	210.162 ± 59.802	188.519 ± 61.956	0.002
Neutrophil count (10^9^/L)	2.030 (1.445-2.815)	2.005 (1.500-2.808)	0.686
Monocyte count (10^9^/L)	0.460 (0.360-0.600)	0.400 (0.332-0.520)	0.015
Lymphocyte count (10^9^/L)	1.905 (1.500-2.413)	1.680 (1.330-2.245)	0.016
NLR	1.000 (0.761-1.405)	1.100 (0.907-1.580)	0.016
PLR	105.981 (82.578-133.532)	107.664 (79.910-146.605)	0.982

Data are mean (SD), number (%), or median (25th–75th percentiles) according to the data type and distribution. NLR, neutrophil-to-lymphocyte ratio; PLR, platelet-to-lymphocyte ratio.

### Correlation Between LV Diastolic Dysfunction and other Clinical Parameters in the Overt Hyperthyroid Patients

Univariate logistic regression analyses revealed that age, duration of hyperthyroidism, BMI, SBP, Scr, PLT count, lymphocyte count, and NLR were all positively correlated with LV diastolic dysfunction in the overt hyperthyroidism subjects. A multiple logistic regression analysis revealed age, duration of hyperthyroidism, BMI, neutrophil count, and NLR may be independent risk factors for the development of LV diastolic dysfunction (**Table 3**). Both univariate and multivariate logistic regression analysis revealed that NLR was strongly correlated with LV diastolic dysfunction (OR = 1.457, 95%CI = 1.074-1.976, P = 0.016; OR=11.753, 95%CI = 1.938-71.267, P = 0.007, respectively).

**Table 3:** Univariate and multivariate analysis of risk factors associated with LV diastolic disfunction in overt hyperthyroid subjects.

	Simple		Multiple	
	P	OR(95% CI)	P	OR(95% CI)
Age	<0.001	1.096 (1.071-1.122)	<0.001	1.104 (1.059-1.15)
Female	0.988	1.004 (0.615-1.637)	0.701	1.266 (0.38-4.223)
duration of hyperthyroidism	0.003	1.059 (1.020-1.099)	0.011	1.089 (1.02-1.162)
BMI	<0.001	1.212 (1.105-1.329)	<0.001	1.329 (1.158-0.526)
SBP	0.036	1.017 (1.001-1.032)	0.072	1.027 (0.998-1.057)
DBP	0.726	0.997 (0.979-1.015)	0.133	0.977 (0.947-1.007)
Scr	0.041	1.017 (1.001-1.033)	0.683	1.009 (0.967-1.053)
SUA	0.873	1.000 (0.997-1.003)	0.818	0.999 (0.993-1.005)
FT3	0.155	0.988 (0.972-1.005)	0.422	1.012 (0.983-1.043)
FT4	0.294	0.995 (0.987-1.004)	0.851	0.998 (0.979-1.018)
TSH	0.161	0.684 (0.402-1.163)	0.495	0.693 (0.241-1.988)
TPOAb	0.276	1.001 (1.000-1.002)	0.747	1.000 (0.998-1.002)
TgAb	0.155	1.000 (1.000-1.000)	0.376	1.000 (1.000-1.001)
TRAb	0.989	1.000 (0.983-1.018)	0.822	1.004 (0.97-1.039)
WBC count	0.284	0.922 (0.795-1.010)	0.278	1.662 (0.664-4.161)
PLT count	0.003	0.994 (0.990-0.998)	0.287	1.008 (0.993-1.024)
Neutrophil count	0.932	0.991 (0.801-1.226)	0.027	0.148 (0.027-0.801)
Lymphocyte count	0.026	0.682 (0.486-0.956)	0.843	1.036 (0.730-1.470)
NLR	0.016	1.457 (1.074-1.976)	0.007	11.753 (1.938-71.267)
PLR	0.982	1.000 (0.995-1.005)	0.101	0.98 (0.956-1.004)

Data are presented as mean ± standard deviation.

### The Prevalence of LV Diastolic Dysfunction in the Overt Hyperthyroidism Patients Categorized According to Differential NLR

**Figure 2:**
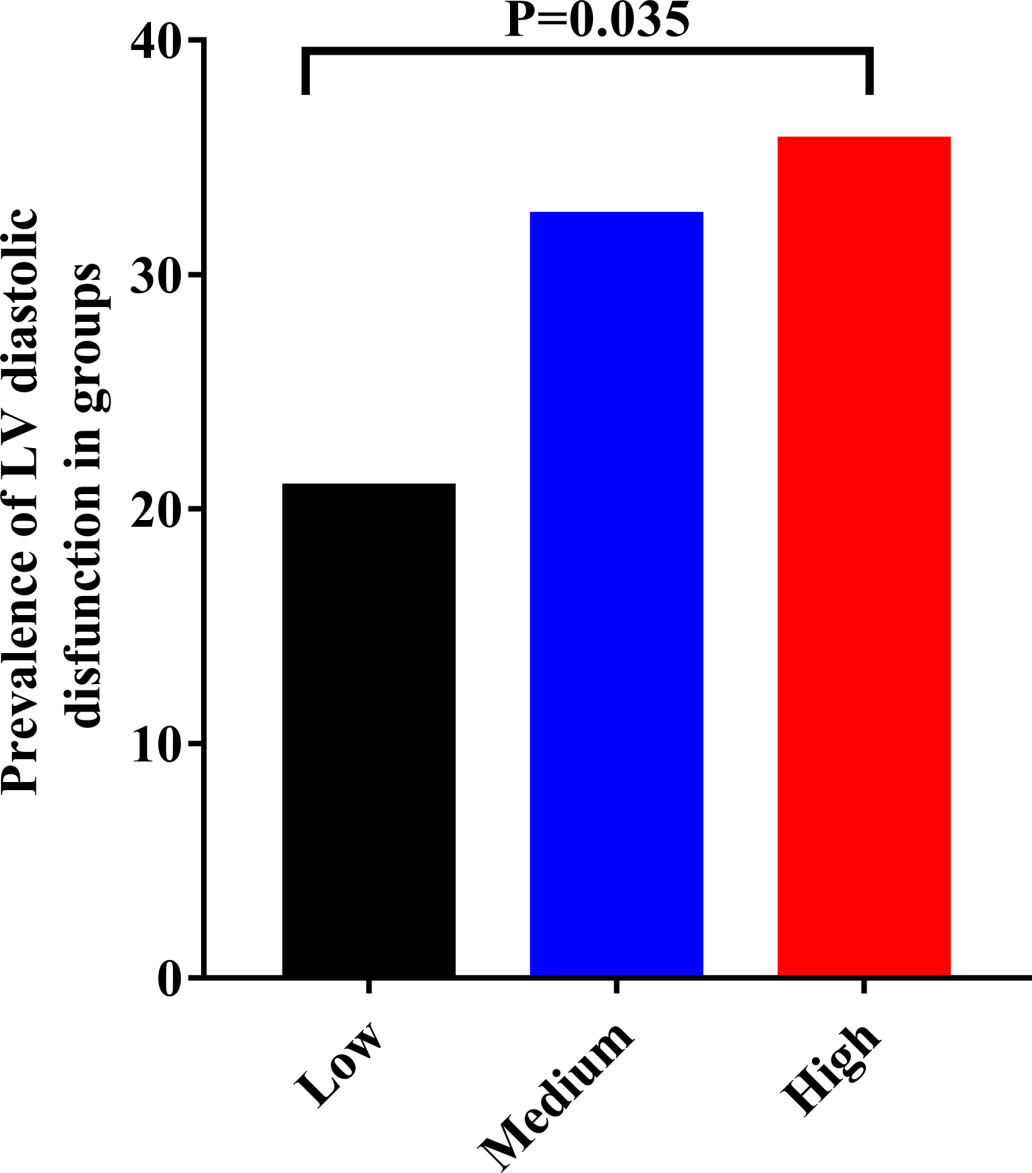
The relationship between different NLR levels and the prevalence of LV diastolic dysfunction in patients with the overt hyperthroidism. Low = NLR<0.879 (n = 115); Medium = 0.879< NLR<1.287(n =120); High = NLR >1.287(n = 115).

According to admission NLR value,350 subjects were divided into tertiles. NLR<0.879 (n = 115), 0.879< NLR<1.287 (n =120), NLR >1.287 (n = 115) were defined as the low, medium, and high NLR level groups, respectively. Among the patients with overt hyperthyroidism, the prevalence of LV diastolic dysfunction in the low, medium and high level groups were 20.9%, 32.5% and 35.7%, respectively (**Figure 2**). The results suggested that as NLR levels increased, the prevalence of LV diastolic dysfunction increased accordingly.

### Characteristics and Laboratory Test Results of Overt Hyperthyroid Patients with Different NLR Levels

The demographic features and laboratory test results of the overt hyperthyroid patients in the three NLR level groups are presented (**Table 4**). Gender, duration of hyperthyroidism, SBP, DBP, PLT count, SUA, TSH, TPOAb, and TgAb were found no statistically significant differences. Age, NLR, BMI, WBC count, neutrophil count, lymphocyte count, Scr, FT3, FT4, and TRAb were statistically significant. Spearson correlation analysis of thyroid hormone and other clinical parameters was presented (**Supplementary Table 1**). Age,BMI,Scr,Neutrophil count and NLR were negative correlation with FT3,but neutropenia/agranulocytosis,Impaired liver function,SBP,SUA,TRAb,monocyte count and lymphocyte count were positive correlation with FT3.Female, Scr, WBC count, Neutrophil count, and NLR were a negative correlation with FT4, but age, neutropenia/agranulocytosis, SBP, TRAb, and monocyte count were a positive correlation with FT4.Scr was negative correlation with TSH, but age, female, and SBP were positive correlation with TSH.

**Table 4:** Demographics, characteristics, and laboratory test results of patients with different NLR levels.

variable	Low (n=115)	Medium (n=120)	High (n=115)
NLR	0.666 (0.521-0.782)	1.039 (0.949-1.137)^*^	1.720 (1.434-2.007)^*^
age (years)	32 (24-46)	37 (29-47)	43 (31-49)^*^
Female, n (%)	81 (70.4%)	80 (66.7%)	73 (64.0%)
duration of hyperthyroidism (years)	1.00 (0.17-3.00)	1.00 (0.17-6.00)	3 (0.08-8.00)
BMI (kg/m2)	19.78 (18.67-21.48)	20.94 (19.05-22.74)^*^	20.70 (19.16-22.76)^*^
SBP (mmHg)	130 (119-137)	126 (113-134)	128 (116-140)
DBP (mmHg)	76 (71-83)	76 (71-83)	76 (64-85)
WBC count (10^9^/L)	4.03 (3.38-5.41)	4.62 (3.72-5.77)^*^	4.96 (3.90-5.93)^*^
PLT count (10^9^/L)	200 (150-241)	211 (174-243)	200 (160-243)
Neutrophil count (10^9^/L)	1.43 (1.05-1.83)	2.10 (1.68-2.63)^*^	2.60 (2.12-3.39)^*#^
Lymphocyte count (10^9^/L)	2.20 (1.70-2.90)	2.02 (1.61-2.44)	1.60 (1.24-1.92)^*#^
Scr (mg/dl)	38.0 (31.8-45.9)	42.9 (37.0-53.7)^*^	45.2 (36.2-60.9)^*^
SUA (umol/L)	306.4 (270.4-353.2)	312.4 (260.1-362.1)	310.3 (252.1-360.9)
FT3 (pmol/L)	31.5 (21.6-46.1)	25.2 (16.0-39.0)^*^	21.2 (11.7-30.4)^*#^
FT4 (pmol/L)	82.1 (49.9-100.0)	67.2 (46.4-100)	47.9 (33.6-82.9)^*#^
TSH (mIU/ml)			
<0.005	89 (77.4%)	77 (64.2%)	85(73.9%)
≥0.005	26 (22.6%)	43 (35.8%)	30(26.1%)
TPOAb (IU/ml)	195.0 (42.9-517.5)	183.9 (39.8-400.3)	215.3(73.7-386.7)
TgAb (IU/ml)	62.1 (14.87-332.2)	127.2 (13.6-783.3)	87.9(15.9-617.1)
TRAb (IU/L)	20.6 (6.91-33.5)	10.4 (4.7-18.6)^*^	10.4(4.49-23.17)^*^

*represents P < 0.05 (medium and/or high group compared with low group respectively).

#represents P < 0.05 (comparison between high and medium group).

## Discussion

In this retrospective study, we explored the association between NLR and LV diastolic dysfunction in overt hyperthyroid patients. Our study revealed that NLR values are independently associated with LV diastolic dysfunction in subjects with overt hyperthyroid in a dosage-related manner. The results suggested that NLR may be used as an auxiliary indicator in the early diagnosis of LV diastolic dysfunction in the overt hyperthyroid subjects.

Thyroid hormone acts in several manners on the heart and peripheral vasculature.A significant proportion of patients with heart failure present some form of thyroid dysfunction including hypothyroidism, hyperthyroidism, and low T3 syndrome (17). Subclinical changes in LV structure and function might be related to the vital clinical outcomes even in patients with subclinical hypothyroidism or hyperthyroidism (18, 19). A previous study showed TSH concentration was not associated with LV structure in either sex, but was inversely related to LV contractility, which was consistent with the known inotropic effects of thyroid hormone (19). Moffat et al. (20) showed the important link between thyroid disease and increased cardiovascular morbidity and mortality may not necessarily be due to abnormal thyroid hormones, but may be mediated through other mechanisms, such as chronic systemic inflammation caused by autoimmunity and/or triggered by ^131^I treatment. Our study showed the value of E/A is low in the patients with LV-diastolic dysfunction, compared with the patients with no LV diastolic dysfunction. The value of E/A is decreased by the LV-diastolic dysfunction. It reminds us that impaired myocardial relaxation (16) may have developed in the overt hyperthyroidism, so we need to pay more attention to the LV-diastolic dysfunction. Our results showed that serum FT3 and FT4 levels were lower in patients with diastolic dysfunction, but the difference was not significant. TSH concentration was not associated with LV diastolic dysfunction. It reminds us that chronic systemic inflammation may play a great role in the development of LV diastolic dysfunction in patients with overt hyperthyroidism.

Recent studies showed that anaplastic thyroid carcinoma(ATC) patients whose NLR increased had worse prognosis than non-increased patients (21). NLR is also considered a novel potential biomarker of higher inflammation severity in thyroid ophthalmopathy (22). Erkan et al. (23) demonstrated that NLR was increased in patients with symptomatic intermediate carotid artery stenosis and the elevated NLR value was an independent variable for carotid artery plaques to become symptomatic. Many researches showed NLR was a biomarker of cardiovascular risk and adverse cardiovascular prognosis in peritoneal dialysis patients younger than 60 years old (23–26). In patients with terminal heart failure, increased NLR was associated with elevated mortality or heart transplantation risk (27). However, the association between NLR and cardiovascular disease has been little investigated in overt hyperthyroid patients. Murat et al. (28) found that NLR did not increase in hyperthyroid patients and this ratio decreased due to the decrease in neutrophil levels in Graves’ patients and NLR was not a suitable indicator of hyperthyroidism. In our research, there were not significantly different in the incidence of neutropenia or agranulocytosis in overt hyperthyroid patients with or without LV diastolic dysfunction. The reason may be that NLR is not a suitable indicator of hyperthyroidism, but a suitable indicator of LV diastolic dysfunction.

Hyperthyroidism enhances the activity of key cells of the innate immune system (namely neutrophils, macrophages, natural killer cells, and dendritic cells) and the activity and proliferation of important cells of the adaptive immune system (namely B- and T- lymphocytes) (29). Hence, NLR is related to two distinct complementary immune pathways that initiate inflammatory responses. Higher NLR values may represent a predomination of innate immunity, while lower NLR values may represent a predomination of adaptive immunity.

Our study found that higher NLR values were associated with greater odds of LV diastolic dysfunction in subjects with overt hyperthyroidism, which may suggest that innate immunity-induced inflammation may make a critical impact on the development of LV diastolic dysfunction in overt hyperthyroid patients. Previous studies showed TH plays an important role in the innate immune system (30). A recent study found that human neutrophils expressed thyroid hormone receptor­α (TRα) at the transcriptional level (31) and neutrophils contained essential elements required for intracellular TH metabolism and TH action, including TH transporters, deiodinases, and TRα. In general, increased TH levels resulted in an amplification of the pro-inflammatory response of neutrophils. However, disturbances in cellular thyroid hormone metabolism are mainly connatural and include mutations, such as the TRα(thyroid hormone receptor­α) or TRβ(thyroid hormone receptor­β) receptor (32). In the cardiomyocyte, T3 (3,5,3′­triiodothyronine) binds to thyroid hormone receptors in the nucleus, which in turn bind to thyroid hormone response elements in the regulatory regions of target genes to regulate transcription. The two main thyroid receptors are TRα receptor, which is highly expressed in cardiomyocytes (33), and TRβ receptor. TRβ receptor mutations, frequently including amino acid changes in the ligand-binding area not allowing for proper T3 binding to the TR, lead to the condition of resistance to thyroid hormones (34). More recently accumulated evidence indicates that the thyroid TRα products were more predominant in the human heart. It should also be noted that the status of T3 receptor levels in patients with heart failure is currently unclear, because in addition to decreases in TR levels, increases in T3 receptor levels have also been reported (35). Mutations of thyroid hormone receptor α1 (TRα1) caused resistance to thyroid hormone (RTHα). TRα1 mutants could act to cause abnormal heart structure, weaken contractility, and disrupt sarcomere organization that affects heart functions. And previous studies showed that TRαresulted in an increase in circulating IL-8 levels, which has also been described in hyperthyroid patients,and elevated IL-8 is a general feature of TRα. Serum IL-8, a neutrophil-activating cytokine, was higher in patients with resistance to TRα compared to healthy controls (35, 36). Therefore, innate immunity-induced inflammation may play a key role in the overt hyperthyroid patient with LV diastolic dysfunction.

There are also some limitations as follows in this study. First of all, the sample size is too small to illustrate the relationship between NLR and LV diastolic dysfunction in subjects with overt hyperthyroidism, so larger studies are needed for further evaluation. Secondly, our research is a cross-sectional study, so in the future, a prospective study is required to explore the NLR levels after getting therapy or with further deterioration of LV diastolic dysfunction. Tertiary, due to the limitations of ultrasound technology and measurements at the time, LV diastolic dysfunction by modalities was still insufficient to assess in this study, we believe that this theoretical bias has a limited impact on the results. And we are unable to obtain sufficient and sufficient data in the diagnosis and classification of left ventricular diastolic dysfunction. In addition, other common inflammatory factors and immune mediators were not measured in our study.

In summary, our findings demonstrated that NLR is strongly associated with LV diastolic dysfunction in subjects with overt hyperthyroidism and that the prevalence of LV diastolic dysfunction is positively correlated with NLR levels. Our research demonstrated that NLR may be used as an auxiliary predictor of LV diastolic dysfunction in patients with overt hyperthyroidism and may be applied in clinical practices to assist physicians in monitoring the development and progression of LV diastolic dysfunction.

## Data Availability Statement

The raw data supporting the conclusions of this article will be made available by the authors, without undue reservation.

## Ethics Statement

This study complied with the Declaration of Helsinki and was approved by the Ethics Committee of Tongji Medical College, Huazhong University of Science and Technology. The patients/participants provided their written informed consent to participate in this study.

## Author Contributions

JZ, LW, and YL conceived and supervised the study. HL and HZ collected the epidemiological and clinical data. HZ, HL, JZ, and YB summarized the data. HZ did data analysis and draft the manuscript. JZ, LW, and YL revised the final manuscript. All authors contributed to the article and approved the submitted version.

## Funding

Funding to support this survey was provided by the Natural Science Foundation of Hubei province (2013CFB091) which was funded by the Science and Technology Department of Hubei.

## Conflict of Interest

The authors declare that the research was conducted in the absence of any commercial or financial relationships that could be construed as a potential conflict of interest.

## Publisher’s Note

All claims expressed in this article are solely those of the authors and do not necessarily represent those of their affiliated organizations, or those of the publisher, the editors and the reviewers. Any product that may be evaluated in this article, or claim that may be made by its manufacturer, is not guaranteed or endorsed by the publisher.
