# Supplementary material for: Neutrophil-to-lymphocyte Ratio is Associated with LV Diastolic Dysfunction in the Overt Hyperthyroid Patients
Source: Front Endocrinol (Lausanne). 2022 Jul 14;13:906947. doi: 10.3389/fendo.2022.906947 (PMC9329826; doi:10.3389/fendo.2022.906947)
Supplement: Supplementary file 1 [file Table_1.doc]

Supplementary table 1 **|** Spearson Correlation Analysis of Thyroid Hormone and Other Clinical Parameters.

|  | FT3 | |  | FT4 | |  | TSH | |
| --- | --- | --- | --- | --- | --- | --- | --- | --- |
|  | Rs | P-value |  | Rs | P-value |  | Rs | P-value |
| age | -0.217 | ＜0.001 |  | 0.181 | 0.001 |  | 0.155 | 0.004 |
| Female | 0.098 | 0.068 |  | -0.187 | ＜0.001 |  | 0.167 | 0.002 |
| duration of hyperthyroidism | -0.053 | 0.323 |  | 0.011 | 0.842 |  | -0.013 | 0.811 |
| Positive family history thyroid disease(n,%) | 0.002 | 0.967 |  | -0.027 | 0.613 |  | 0.011 | 0.840 |
| Neutropenia/Agranulocytosis(n,%) | 0.189 | ＜0.001 |  | 0.204 | ＜0.001 |  | 0.005 | 0.927 |
| Impaired liver function(n,%) | 0.015 | 0.039 |  | 0.063 | 0.258 |  | -0.038 | 0.495 |
| BMI | -0.151 | 0.009 |  | -0.105 | 0.069 |  | 0.046 | 0.420 |
| SBP | 0.116 | 0.034 |  | 0.136 | 0.013 |  | 0.148 | 0.006 |
| DBP | 0.057 | 0.296 |  | 0.055 | 0.314 |  | 0.023 | 0.678 |
| Scr | -0.399 | ＜0.001 |  | -0.441 | ＜0.001 |  | -0.264 | ＜0.001 |
| SUA | 0.142 | 0.01 |  | 0.064 | 0.252 |  | -0.082 | 0.142 |
| TPOAb | 0.027 | 0.631 |  | 0.010 | 0.862 |  | -0.028 | 0.621 |
| TgAb | 0.062 | 0.278 |  | 0.098 | 0.086 |  | 0.003 | 0.959 |
| TRAb | 0.431 | ＜0.001 |  | 0.374 | ＜0.001 |  | 0.044 | 0.420 |
| WBC count | -0.072 | 0.182 |  | -0.121 | 0.024 |  | -0.013 | 0.813 |
| PLT count | 0.058 | 0.283 |  | -0.026 | 0.630 |  | -0.036 | 0.500 |
| Neutrophil count | -0.211 | ＜0.001 |  | -0.219 | ＜0.001 |  | -0.008 | 0.886 |
| Monocyte count | 0.177 | 0.001 |  | 0.113 | 0.034 |  | 0.003 | 0.951 |
| Lymphocyte count | 0.112 | 0.037 |  | 0.046 | 0.395 |  | -0.019 | 0.721 |
| NLR | -0.271 | ＜0.001 |  | -0.220 | ＜0.001 |  | -0.030 | 0.577 |
| PLR | -0.074 | 0.171 |  | -0.079 | 0.142 |  | -0.017 | 0.744 |
